# New perspectives for motivating better decisions in older adults

**DOI:** 10.3389/fpsyg.2015.00783

**Published:** 2015-06-22

**Authors:** JoNell Strough, Wändi Bruine de Bruin, Ellen Peters

**Affiliations:** ^1^Department of Psychology, West Virginia University, Morgantown, WV, USA; ^2^Centre for Decision Research, Leeds University Business School, University of Leeds, Leeds, UK; ^3^Department of Engineering and Public Policy, Carnegie Mellon University, Pittsburgh, PA, USA; ^4^Department of Psychology, The Ohio State University, Columbus, OH, USA

**Keywords:** motivation, decision making, aging, interventions, mental models, self efflicacy, emotions, cognition

## Abstract

Decision-making competence in later adulthood is affected by declines in cognitive skills, and age-related changes in affect and experience can sometimes compensate. However, recent findings suggest that age-related changes in *motivation* also affect the extent to which adults draw from experience, affect, and deliberative skills when making decisions. To date, relatively little attention has been given to strategies for addressing age-related changes in motivation to promote better decisions in older adults. To address this limitation, we draw from diverse literatures to suggest promising intervention strategies for motivating older recipients’ motivation to make better decisions. We start by reviewing the life-span developmental literature, which suggests that older adults’ motivation to put effort into decisions depends on the perceived personal relevance of decisions as well as their self-efficacy (i.e., confidence in applying their ability and knowledge). Next, we discuss two approaches from the health intervention design literature, the mental models approach and the patient activation approach, which aim to improve motivation for decision making by improving personal relevance or by building self-efficacy or confidence to use new information and skills. Using examples from these literatures, we discuss how to construct interventions to motivate good decisions in later adulthood.

## Introduction

Because the global population is aging (Kinsella and He, 2008), it is becoming increasingly important to understand how decision-making competence changes across the adult life span. Aging is associated with cognitive declines (e.g., working memory, fluid intelligence) that can undermine decision-making competence (for reviews, see Del Missier et al., 2015; Zaval et al., 2015). However, age-related changes in emotional skills and experience sometimes compensate for cognitive declines (Li et al., 2014; for reviews, see Peters and Bruine de Bruin, 2012; Strough et al., 2015; Zaval et al., 2015).

In this review, we note that recent research on aging and decision making highlights the potential importance of *motivation* in older adult decision making. Here, motivation is the willingness to engage with a decision, including considering the presented information, deliberating about the options, and selecting the option that is most likely to lead to preferred outcomes. Motivation has been shown to affect the extent to which older adults use experience, affect, and deliberative skills to make decisions (Strough et al., 2011a,b, 2015; Hess and Queen, 2014; Bruine de Bruin et al., 2015). However, relatively little attention has been given to strategies for inducing motivation to promote better decisions in older adults. We therefore draw on diverse literatures to suggest potentially promising intervention strategies for motivating good decision making in older adults.

First, we present findings from the life-span developmental literature on aging and decision making, focusing especially on the role of age-related changes in motivation. Specifically, we note that older adults’ motivation to put effort into decisions depends on the perceived relevance of the presented information as well as on their self-efficacy or confidence in applying their ability and knowledge. Next, we discuss two approaches from the health intervention literature which aim to motivate better health decision outcomes. First, we discuss the mental models approach to developing interventions, which aims to present decision-relevant information for specific audiences, and, if needed, to provide training in applying specific skills. Next, we discuss the patient activation literature, which aims to improve health decisions by boosting patients’ self-efficacy in improving their health and health behaviors. Using examples from these literatures, we discuss how to construct interventions to motivate good decisions in later adulthood.

## Age-Related Changes in Motivation

In this section, we discuss two ways in which older adults may experience changes in their motivation to make decisions. First, we discuss age-related changes in the motivation to limit cognitive effort by focusing on information and decisions that are deemed most relevant. Specifically, we review research that has investigated the role of personal relevance for aging and decision making, much of which has focused on emotional relevance. Second, we discuss age-related changes in self-efficacy or confidence in applying ability and knowledge when making decisions. To date, self-efficacy and confidence have received far less attention in research on aging and decision making compared to work on personal relevance, but work from other literatures suggests these constructs may play an important role in older adult’s decisions.

### Age-Related Changes in the Role of Personal Relevance

Perhaps due to age-related declines in fluid cognitive ability, cognitive processing becomes more costly with age such that older adults become more selective about how to allocate their efforts (Hess, 2014; Hess and Queen, 2014). Specifically, older adults work harder on tasks with high self-relevance compared to those with lower self-relevance (Ennis et al., 2013). This selectivity is viewed as adaptive because it allows older adults to conserve their more limited cognitive resources while maintaining performance in domains that are important to them (Hess, 2014).

However, one potential consequence is that unless tasks are personally relevant, older adults will limit their cognitive effort. For example, older adults request less information to make decisions (Leventhal et al., 1993), examine less available information (Johnson, 1990), and prefer to consider fewer choice options (Reed et al., 2008; von Helversen and Mata, 2012). Such effects may be due not only to age-related cognitive declines, but also to perceived declines, and corresponding age-related decline in motivation to expend cognitive resources for less personally-relevant tasks.

To motivate older adults to apply more of their cognitive resources, it may be important to increase the perceived relevance of decisions. For example, relative to younger adults, older adults’ memory is more likely to be enhanced when personal accountability is high than when it is low (Hess et al., 2001, 2009). Additionally, when older adults have more personal interest in the decision to be made, they take more time to systematically analyze options before making a decision (Meyer et al., 2007). For instance, older adults use more effortful systematic search strategies to review information when self-relevance is high, but less effortful “satisficing” strategies when self-relevance is low; younger adults’ search strategies are less influenced by self-relevance (Hess et al., 2013).

For older adults, personal relevance often centers on the emotional meaningfulness of an activity or task. Socioemotional selectivity theory posits that because older adults increasingly recognize that they have limited time left to live, they become more motivated to optimize positive emotional experiences in the “here and now” (Carstensen, 2006). By contrast, younger people’s expansive time horizons is posited to motivate them to seek a wider variety of new experiences (Carstensen, 2006). Age differences therefore emerge in preferences for social partners, with older adults seeking emotionally close companions who make them feel good and younger adults seeking companions who introduce variety and novelty (Fung et al., 1999). Interestingly, these preferences reverse when older adults imagine an expansive future and young adults, a limited one (Fung et al., 1999). Older adults’ selection of activities that are emotionally meaningful may explain why aging is associated with experiencing emotions that are generally more positive than negative (Charles and Carstensen, 2010; Carstensen et al., 2011).

Older adults’ motivation to seek emotionally meaningful experiences is also presumed to drive increases in preferences for positive relative to negative information (Carstensen and Mikels, 2005; Mather and Carstensen, 2005). Older adults prefer advertisements that target emotion-oriented goals pertaining to loved ones instead of discovery-oriented goals pertaining to future personal success, whereas younger adults’ preferences and memory are unaffected by this distinction (Fung and Carstensen, 2003). When older adults are asked to imagine an extension of their time left in life, they act like younger adults and no longer prefer advertisements that target emotionally-meaningful goals over advertisements that target discovery-oriented informational goals (Fung and Carstensen, 2003). Thus, future time perspective is an important underlying mechanism of preferences for emotionally-meaningful goals and positive relative to negative information.

Older adults’ focus on optimizing positive emotions also affects their responses to decision-relevant information. For example, they rate health pamphlets as more informative when health goal framing is positive, emphasizing benefits of engaging in a behavior, rather than negative, emphasizing negative consequences of not engaging in the same behavior (Shamaskin et al., 2010). When making choices, older adults review and remember relatively more positive than negative information as compared to younger adults (Mather et al., 2005; Löckenhoff and Carstensen, 2007). Presumably, such a selective focus on positive information may help or hurt decisions, depending on the extent to which understanding the negative information is important for the decision at hand (see Reed et al., 2014b; Mikels et al., 2015). When viewing informational videos that include negative images of skin cancer, older adults look less at the material than younger adults and subsequently fail to distinguish melanoma from normal moles (Isaacowitz and Choi, 2012). Even so, older adults are more likely than younger adults to take protective measures such as selecting a sunscreen with a higher SPF (Isaacowitz and Choi, 2012). These findings suggest that older adults may be able to extract relevant information even without attending as much to negative images, although they may also be more risk averse than younger adults.

Older adults’ selective focus on positive information can be reduced by changing their motivation. When decisions have high (versus low) stakes, older adults are more likely to thoroughly review both positive and negative information (Reed et al., 2014b). Directly encouraging older adults to review all information also eliminates age differences in viewing positive or negative healthcare information (Löckenhoff and Carstensen, 2007). Cuing cognitive strategies by having older adults calculate expected value improves the consistency of hypothetical monetary gambles across gain and loss frames, as does asking older adults to use “critical thinking” instead of “gut feelings” (Thomas and Millar, 2012). Indeed, a meta-analysis shows that older adults’ preference toward processing positive information is less apparent when task instructions promote specific processing strategies. Conversely, older adults’ positivity bias is more apparent in naturalistic settings when they are free to pursue their own goals (Reed et al., 2014a).

However, the effectiveness of prompting older adults to focus on information instead of relying on emotional reactions is mixed. When older adults are asked to choose the healthcare plan with the greatest number of positive attributes, instructing them to a focus on “specific details” actually leads to worse decisions than encouraging a focus on “emotional reactions” or providing no instructions at all (Mikels et al., 2010).

Meanwhile, it should be noted that older adults’ motivation to maintain positive emotions can sometimes contribute to better decisions. Older adults’ motivation and coping skills for maintaining positive emotional states are thought to partly explain why they are better than younger adults at making decisions that involve “sunk costs” or lost investments (Strough et al., 2011b; Bruine de Bruin et al., 2014). For example, older adults are more likely than younger adults to stop watching a boring movie or working on an unrewarding hobby irrespective of how much time or money they already “sunk” into the effort (Strough et al., 2008; Bruine de Bruin et al., 2012). According to theories of rational decision making, older adults’ tendency to discontinue failing investments is accurate, because prior losses remain irrecoverable independent of how one proceeds (Arkes and Blumer, 1985).

Other work shows that older adults earn more rewards than younger adults on a two-choice reinforcement learning task where each choice is associated with variable rewards (Worthy et al., 2015). Older adults earn more rewards because they are more likely than younger adults to switch choices following negative feedback about their choice (Worthy et al., 2015). Avoiding negative feedback could facilitate maintaining positive emotional states. In addition, older adults tend to focus more on positive and less on negative attributes of products than younger adults, and they are ultimately more satisfied with the products they choose to take home (Kim et al., 2008). Because satisfaction is often used as a measure of decision quality (e.g., Wilson and Schooler, 1991), these results can be interpreted as meaning that older adults made better decisions than younger adults. Together, these studies suggest that age differences in motivation to maintain positive emotions and associated affect regulation strategies are a pathway that may facilitate objective and subjective facets of good decision making in later adulthood.

### Age-Related Changes in Self-Efficacy or Confidence in Applying Ability and Knowledge

Here, we posit that motivation to engage in decision making may be higher among people who believe they are better equipped to make good decisions. We discuss age differences in confidence to apply one’s ability and knowledge and focus especially on decision-making self-efficacy, self-efficacy related to numbers, memory self-efficacy, and research that has investigated links with decision making. Theories of goal-directed behavior suggest that task choice, persistence, and performance are driven by perceptions of self-efficacy, which refers to beliefs about how well one can perform a task (Bandura, 1997). People who have high self-efficacy believe that they can execute task-relevant skills, leading them to take control of their actions and achieve their goals. Self-efficacy is central to theories of health behavior (see Noar, 2005, for a review) and achievement motivation (see Eccles and Wigfield, 2002; Eccles, 2009; Elliot et al., 2010, for reviews). Hence, self-efficacy likely is relevant to older adults’ decision-making competence.

#### Decision-Making Self-Efficacy

Only a few studies have investigated age differences in decision-making self-efficacy, and they show mixed results. Older adults rate their general decision-making competence as lower than younger adults do, which may reflect older adults’ concerns about cognitive aging (Bruine de Bruin et al., 2012). Older adults also report lower self-efficacy than middle-aged and younger adults, regarding healthcare and daily life decisions (Woodward and Wallston, 1987). Such age-difference findings are important because a composite measure of decision-making self-efficacy beliefs, perceived experience, and need for support when making decisions predicted worse comprehension and use of decision-relevant information in participants aged 25–97 years old (Finucane and Gullion, 2010). Hence, some research suggests that older adults’ lower decision-making self-efficacy could have consequences for their decisions.

However, age differences in decision-making self-efficacy seem to disappear when people are asked how good they are at making “the best” decision in a specific context (Finucane and Gullion, 2010). Some studies even find that older age is associated with higher expectancies about the ability to make “the best” decision (Löckenhoff and Carstensen, 2007; Reed et al., 2013). When decision-making self-efficacy is measured by asking participants to assess their confidence in being able to make the “best” decision, however, it does not predict the type of information people review or remember (Löckenhoff and Carstensen, 2007), older adults’ preferences for fewer choice options compared to younger adults (Reed et al., 2013), or the association between older age and worse decision-making competence (measured as the ability to comprehend information, weigh it appropriately, and overcome impulsive responses; Finucane and Gullion, 2010). Possibly, when thinking about what they would do to make the “best” decision, older adults may imagine committing more of their cognitive effort—even if that is not actually what they end up doing when faced with the decision. It may also be the case that older adults’ definitions of what constitutes the best decision are different from what researchers believe is the best decision. For instance, an older adult may define the “best” decision as one that is “good enough” given motivations to limit effort or does not induce regret whereas researchers may define the best decision as one that has the highest rating on a specific dimension.

#### Number-Related Self-Efficacy

Self-efficacy with respect to specific skills underlying decisions, such as numeric ability, can also be important. Individuals with lower versus higher perceptions of their own numeric ability were less motivated to use numbers and/or less confident in their use in decision-related tasks (Peters and Bjalkebring, 2015). For example, in one numeric memory task, those lower in perceived numeracy were more likely not to provide a recalled number than those higher (controlling for other numeric abilities and general intelligence measures). Paradoxically, those higher in perceived numeracy were somewhat more likely to provide incorrect responses, as if their “hubris” misled them. It appeared as if lower subjective numeracy individuals were less motivated to try to remember the numbers or they had less confidence that their memories were correct relative to those higher in perceived numeracy. Individuals who perceived themselves as higher in subjective numeracy also reacted more positively to numeric gambles, finding them more attractive than those lower in subjective numeracy (Peters and Bjalkebring, 2015).

Consistent with the finding that individuals with high subjective numeracy respond more positively to numeric tasks, Miron-Shatz et al. (2014) demonstrated that higher subjective numeracy (but not objective numeracy) was associated with a greater willingness to pay for direct-to-consumer genetic testing results. One explanation of this finding is that subjective numeracy indirectly influenced the perceived value of these highly numeric test results through negative emotional reactions and a lack of motivation to receive probabilistic information. In other studies, those higher in subjective (but not objective) numeracy expressed greater preferences for providing and receiving numeric information rather than just words in health communications (Couper and Singer, 2009; Anderson et al., 2011). These findings are important because older adults score lower than younger adults on measures of objective and subjective numeracy (Peters et al., 2007; Reyna et al., 2009; Smith et al., 2010; Rolison et al., 2013; Bruine de Bruin et al., 2015). A recent study suggested that these age declines may be due to age declines in motivation to think hard about complex tasks. Need for cognition, which is defined as intrinsic motivation to exert cognitive effort (Cacioppo et al., 1996) mediated age declines in objective numeracy (Bruine de Bruin et al., 2015).

#### Memory Self-Efficacy

Memory self-efficacy may be another domain-specific set of beliefs important for motivating good decisions because memory is a skill that is associated with better performance on a variety of decision-making tasks (Del Missier et al., 2013, 2015). The perception of having better memory skills may influence the motivation to approach situations that require remembering complex information (Berry, 1999), and, hence, could potentially be important for motivating good decision making in older adults. Yet, few studies have been conducted on this topic. There is evidence that older adults who hold more positive beliefs about their memory prefer life-sustaining treatment (Allen et al., 2011). However, perceptions of memory ability do not explain why older adults prefer fewer choice options than younger adults do (Reed et al., 2013).

The reasons for age differences in memory self-efficacy have also been explored. One obvious reason may be older adults’ awareness of objective age-related changes in memory (see Hess, 2005, for a review), but older adults’ lower self-efficacy beliefs may also reflect pervasive negative cultural stereotypes about cognitive aging (Hummert, 2011). Activating these stereotypes can have negative consequences for older adults’ memory performance (see Barber and Mather, 2014, for a review). Importantly, part of the reason that memory interventions are successful is because they increase recipients’ self-efficacy beliefs about the quality of their memory (West and Hastings, 2011). Indeed, recent work suggests that memory self-efficacy beliefs predict the extent to which recipients benefit from interventions to improve more general fluid cognitive abilities (Payne et al., 2012).

#### Confidence in Knowledge

Research on under/overconfidence demonstrates that people tend to be overconfident (they express more confidence) in their knowledge than is warranted by their performance on knowledge tasks (Keren, 1991). Although the validity of overconfidence as a construct has been questioned (Gigerenzer et al., 1991; Juslin et al., 2000), having more accurate confidence in knowledge has been associated with better decision processes and with achieving better life decision outcomes (Bruine de Bruin et al., 2007b). Moreover, being more confident than is justified by one’s knowledge may have positive or negative effects on decision quality depending on the situation (Parker and Stone, 2014). Being overconfident may be beneficial in some domains because it may allow people to perceive themselves as having more self-efficacy to act. Indeed, having more confidence in retirement planning knowledge than is warranted by actual knowledge predicts engaging more in retirement planning and avoiding unnecessary fees in hypothetical investment tasks (Parker et al., 2012).

Mixed evidence exists about age differences in confidence in knowledge. Some studies show that older adults are better than younger adults at recognizing the limitations of their decision-relevant knowledge (Kovalchik et al., 2005; Bruine de Bruin et al., 2012), but there is also evidence that older adults are more overconfident than younger adults (Crawford and Stankov, 1996); still other studies find that older adults are similar to younger adults (Hansson et al., 2008). Whether age differences emerge may partly reflect the cognitive demands of the task at hand, with older adults being more likely to overestimate how much they know when assessing confidence itself is cognitively demanding (Hansson et al., 2008). Moreover, perceived stereotypes about age-related cognitive decline have been shown to affect changes in memory performance with age (e.g., Levy et al., 2012).

## Intervention Strategies for Motivating Better Decisions

In this section, we discuss two types of intervention strategies for motivating good decisions by building on evidence reviewed earlier about personal relevance and self-efficacy. The first strategy type is based in the mental models approach, which aims to motivate better decisions by presenting information that is deemed relevant by recipients. The second is the patient activation approach, which is linked theoretically with motivating better decisions by improving self-efficacy beliefs.

### Mental Models Approach Toward Developing Interventions

The mental models approach aims to develop interventions that present information relevant to the specific decisions faced by specific audiences. It is grounded in science education, health communications, cognitive anthropology and psychology, which have suggested that people search and interpret new information in light of their existing beliefs, also referred to as “mental models” (e.g., Meyer et al., 1985; Nersessian, 1992; Gentner, 2002; Morgan et al., 2002; Bruine de Bruin and Bostrom, 2013). For example, education research has found that a child who believes that the earth is flat may interpret new information that the earth is round as suggesting that the earth is round like a flat pancake (Vosniadou, 2002). Teachers should therefore focus on showing children why the horizon appears flat when the earth is actually round (Vosniadou and Brewer, 1992). Hence, “mental models” communication strategies aim to provide information that recipients can understand and apply to their own experiences.

Here, we focus on the mental models approach for developing risk communication, which seeks to inform people’s decisions about risks. We first provide a description of the mental models approach, and the process it uses to design interventions that recipients deem relevant. Although the mental models approach has not been applied to developing communications for older adults, we discuss its potential for addressing age-related changes in the motivation to engage more selectively with relevant information.

#### Description of Mental Models Approach

To design interventions that recipients deem relevant, the mental models approach recommends intervention development in three systematic steps. First, the normative step aims to identify how decisions should be made to achieve the best outcomes for recipients. The scientific literature is consulted to identify the factors that lead to the best decision outcomes, while recognizing that individuals may vary in which decision outcomes they prefer. Second, the descriptive step aims to understand how people actually make their decisions, and whether they want or need help to make better decisions. The goal is to identify strengths and weaknesses in decision making, whether real or perceived. To this end, interviews and surveys are conducted with members of the intended audience, often augmented with observational and experimental methods that do not rely on self-reports (Bruine de Bruin and Bostrom, 2013). Third, the prescriptive step involves initial intervention design, aiming to address weaknesses while building on strengths, in ways that work best for the specific audience. Attention is given to wording and format. Finally, interventions are tested for effectiveness, in terms of influencing comprehension and helping recipients to achieve their preferred decision outcomes. Hence, the resulting intervention is designed to be relevant to the intended recipients, in terms of addressing their concerns in a format that they can understand and find appealing.

As noted, the mental models approach has not yet been applied to developing communications for older adults. Here, we therefore provide two examples of mental models communication development, while noting implications for applying the mental models approach to develop communications for older adults. The first example is a mental models communication that targeted public concerns about cancer risks from electro-magnetic fields, which was designed to meet the preferences of the intended audience (Morgan et al., 2002). The authors recognized that some recipients wanted to know how to reduce their exposure to electro-magnetic fields, even after learning that the existing scientific evidence did not support their worries about the cancer risks. They therefore responded to this request by (a) explaining that worries were unfounded; (b) mentioning strategies for reducing exposure to electro-magnetic fields without incurring costs (so as to take into account the potential of obtaining no benefits), including avoidance of electric bedding, moving appliances with electric motors (e.g., alarm clocks) away from the bed, and pushing computer monitors further away on one’s desk; and (c) discussing better strategies for reducing cancer risks, such as quitting smoking and eating healthier. Although the final version of this mental models intervention was not evaluated via a randomized-controlled trial, its sales suggested that the intended audience found it useful. Between 1989 and 1995, it sold 150,000 copies.

The second example of a mental models intervention pertains to an interactive video that targeted young women’s sexual decisions (Downs et al., 2004, in press; Bruine de Bruin et al., 2007a). Among other things, the intervention focused on teaching recipients the skills and confidence needed to negotiate risk mitigation with their sexual partners, because interviews with young women indicated that this was one of their main concerns. A randomized-controlled trial found that recipients of the video intervention reduced sexual activity, were more likely to use condoms when sexually active, reported fewer sexually transmitted infections, and were less likely to test positive for Chlamydia on an objective clinical test given 6 months later.

#### Application to Older Adults’ Decisions

To the best of our knowledge, mental models interventions have not yet been designed for older adults. However, the mental models approach may be especially useful for developing interventions for older adults, as it expressly aims to provide information that the intended audience deems relevant and wants to receive. As a reminder, older adults (more than younger adults) appear to be motivated to engage selectively with information that is relevant and fits with their personal goals (e.g., Hess, 2014). For example, interventions could be designed to meet older adults’ preferences for responding to positive information (e.g., the benefits of walking) than for negative information (e.g., the costs of not walking; Notthoff and Carstensen, 2014). Similarly, interventions that build on older adults’ strengths (e.g., emotional skills, life experience) may make them feel more positive, confident, and motivated than interventions that attempt to fix older adults’ weaknesses (e.g., memory, fluid cognition). When using the mental models approach to design interventions targeting older adults’ decisions, the design process would involve qualitative interviews and surveys that would help to highlight older adults’ specific concerns, their preferences for information, and any decision-making deficits in need of intervention. With respect to this latter point, the design process may uncover, for example, the need to highlight negative information that older adults might otherwise ignore.

The mental models approach, thus, encourages intervention designers to go beyond their intuitions of what the intended audience needs. Because intervention designers are domain experts in their field, they often misjudge what information non-experts need or find most compelling (Bruine de Bruin and Bostrom, 2013). Older adults may be perceived by others as lacking the ability, drive, or information to make informed decisions. However, the problem may reside more in the older adults’ perceptions of the relevance of the decision (and why they should engage in it) and less in their actual abilities. People of all ages experience problems when making decisions. Effective interventions are those tailored to helping them overcome whatever problems are most critical to their decision-making success.

### Patient Activation Approach Toward Developing Interventions

To address how to change perceived efficacy, we turn to research on *patient activation*, which recognizes that providing patients the knowledge, skills, and confidence to manage their own health and healthcare will produce better health outcomes (Hibbard and Mahoney, 2010). This research assumes that, to make good decisions, people not only need information, but they also need to have a sense of efficacy that they can follow through on health behaviors and that doing so will be valuable to them. Studies concerning patient activation indicate that patients go through four stages in the process of becoming competent managers of their own health: understanding that they have an important role to play in managing their health; gaining the knowledge and confidence to take action; taking action; and maintaining those behaviors even under stress (Hibbard et al., 2004, 2005). Understanding how to move patients from one activation level to the next may provide insights into how to motivate older adults to consider information they might not otherwise and to apply their decision-making competence, especially at the second stage (of gaining knowledge and confidence to take action).

#### Description of Patient Activation Strategies

The patient activation approach is a pragmatic one developed by health services researchers and built loosely on earlier self-efficacy literature and more recent research in positive psychology (Bandura, 1997; Frederickson, 2001; Isen, 2008; Hibbard and Mahoney, 2010). In correlational data, individuals scoring higher on a patient activation measure (Hibbard et al., 2005) also experience better health outcomes including fewer emergency visits, less obesity, less smoking, and more positive clinical indicators such as blood pressure within normal range (Greene and Hibbard, 2012).

Although most patient activation research has been correlational and, thus, leaves open the possibility that healthier patients are simply more likely to be activated, recent efforts have turned toward interventions, including with older adults. The interventions generally use one of three strategies: First, some interventions have focused on skills development and have found that patient training and support in how to ask questions increased how much patients participated in their own care. Deen et al. (2011), for example, approached patients in community health centers as they waited for their physician visits. The experimenters helped the patients to brainstorm questions, to understand what information might emerge from the questions (including how it might inform decisions that might be made during their visit), and to prioritize questions. They also reminded participants that asking their doctor questions might improve the care they receive. This brief intervention improved patient activation in lower SES individuals although the effects on actual health decisions or outcomes are unknown at this time.

A related strategy emerges from health-efficacy studies (e.g., McAuley et al., 1994). In it, skill building interventions in specific areas of interest are augmented by social support interventions to enhance self-efficacy. In one example, Covey et al. (2012) recruited patients aged 45 years and older with chronic obstructive pulmonary disease and attempted to increase their exercise behaviors. Participants learned strategies to overcome barriers to exercise and to maintain exercise as a healthy life style. They also formed “buddy” groups for support, were given structured feedback from staff and guidance for returning to exercise if it had been halted, and viewed videotapes of other people like themselves progressing through training to facilitate their learning. Although effects were not strong, some evidence existed that the self-efficacy enhancing intervention was beneficial to increasing exercise (see also Larson et al., 2014).

The second intervention strategy focuses on increasing motivation by providing financial incentives which may make the activity more personally relevant and therefore meaningful to older adults. Frosch et al. (2010), for example, assigned one group of older adults to an encouragement condition (they received a gift card if they attended at least three group sessions); a non-encouraged group did not receive a gift card. Encouraged participants were more likely to attend sessions, to report greater activation, and more physical activity and health-related quality of life. However, more research on financial incentives is needed due to mixed results. For example, Kullgren et al. (2014) randomly assigned older adults to receiving a financial incentive, peer support through an online message board, both, or weekly feedback only. Neither financial incentives nor online peer support increased walking behaviors relative to weekly feedback among these older adults.

In a third intervention strategy that has been hypothesized but not specifically tested to the best of our knowledge, support would be tailored to the patient’s current level of activation. In it, small approachable steps would encourage individuals at low activation whereas more challenging behaviors would encourage individuals who are already fairly well activated. The theoretical focus concerns the building of positive experiences and emotions to create increases in self-efficacy beliefs and a positive upward cycle of success toward better health (Hibbard and Mahoney, 2010). The strategy starts with finding out where the person is in terms of knowledge, skills, and confidence with respect to their personal health (i.e., determining the patient’s current activation level), and then giving them the next small step that they need to take. Self-efficacy and success are thought to build from there. Although this idea appears promising, little research has been conducted beyond one paper demonstrating that less activated patients (compared to more activated ones) experienced fewer positive emotions and more negative emotions controlling for socioeconomic indicators; less activated patients also had fewer health goals for themselves (Hibbard and Mahoney, 2010).

#### Application to Older Adults’ Decisions

Some of the strategies in the patient activation approach have been used already with older adults (e.g., Frosch et al., 2010). The patient activation approach may be an especially useful one for developing self-efficacy interventions for older adults, as such efficacy appears to be a requirement for activation to be translated into action. One example of this approach comes from Lorig et al. (2009). They demonstrated in an online diabetes self-management program that asking participants to reply each week to a question such as “What problems do you have because of your diabetes?” and to make a specific action plan was associated with increases in self-efficacy and patient activation as well as a small improvement in hemoglobin A1C levels (other health indicators such as self-reported exercise did not improve, however). The program itself was situated within community online bulletin boards facilitated by two moderators making it unclear how much cognitive versus social engagement mattered to the final outcomes. Understanding this difference may be critical to the best and most efficient efficacy interventions with older adults.

Like the mental models approach, the activation approach encourages intervention designers to go far beyond their intuitions of what older adults need to a descriptive understanding of how to tailor interventions to existing knowledge, confidence, and skills and to build activation from there.

## Conclusion

In summary, recent developments in the life-span developmental literature suggest that age-related changes in *motivation* can affect the extent to which older adults spend effort on making decisions (e.g., Strough et al., 2011a,b, 2015; Bruine de Bruin et al., 2015). Specifically, older adults’ motivation to put effort into decisions appears to change in two ways. First, they become less interested in spending effort on decisions that they perceive as less relevant to achieving their goals (Hess, 2014), with the maintenance of positive emotions growing more relevant with age (Carstensen, 2006). Second, they may feel less confident that they have the ability to make good decisions, at least to the extent that decisions rely on memory (Berry, 1999; Del Missier et al., 2015), numeric ability (Peters and Bjalkebring, 2015), and fluid cognitive ability (Bruine de Bruin et al., 2012). By combining this work with approaches from the health intervention design literature on mental models (e.g., Bruine de Bruin and Bostrom, 2013) and patient activation (Hibbard and Mahoney, 2010), we have identified potentially promising strategies to promote better decisions. Future research is necessary to test the effectiveness of these strategies for motivating better decisions in older adulthood.

### Conflict of Interest Statement

The authors declare that the research was conducted in the absence of any commercial or financial relationships that could be construed as a potential conflict of interest.

## References

[B1] Allen J. Y. Hilgeman M. M. Allen R. S. (2011). Prospective end-of-life treatment decisions and perceived vulnerability: future time left to live and memory self-efficacy. Aging Ment. Health 15, 122–131. 10.1080/13607863.2010.505229 20924818

[B2] Anderson B. L. Obrecht N. A. Chapman G. B. Driscoll D. A. Schulkin J. (2011). Physicians’ communication of Down syndrome screening test results: the influence of physician numeracy. Genet. Med. 13, 744–749. 10.1097/GIM.0b013e31821a370f 21637105

[B3] Arkes H. R. Blumer C. (1985). The psychology of sunk cost. Organ. Behav. Hum. Decis. Process. 35, 124–140. 10.1016/0749-5978(85)90049-4

[B4] Bandura A. (1997). Self-efficacy: The Exercise of Control. New York, NY: Freeman.

[B5] Barber S. J. Mather M. (2014). “Stereotype threat in older adults: when and why does it occur and who is most affected?” in Emotion, Social Cognition, and Problem Solving During Adulthood, eds Verhaeghen P. Hertzog C. (New York, NY: Oxford University Press), 302–319.

[B6] Berry J. M. (1999). “Memory self-efficacy in its social cognitive context,” in Social Cognition and Aging, eds Hess T. M. Blanchard-Fields F. (San Diego, CA: Academic Press), 70–96.

[B7] Bruine de Bruin W. Bostrom A. (2013). Assessing what to address in science communication. Proc. Natl. Acad. Sci. U.S.A. 110, 14062–14068. 10.1073/pnas.1212729110 23942122 PMC3752171

[B8] Bruine de Bruin W. Downs J. S. Fischhoff B. (2007a). “Adolescents’ thinking about the risks and benefits of sexual behavior,” in Thinking with Data, eds Lovett M. C. Shah P. (Mahwah, NJ: Erlbaum), 421–439.

[B9] Bruine de Bruin W. Parker A. M. Fischhoff B. (2007b). Individual differences in adult decision-making competence. J. Pers. Soc. Psychol. 92, 938–956. 10.1037/0022-3514.92.5.938 17484614

[B10] Bruine de Bruin W. McNair S. J. Taylor A. L. Summers B. Strough J. (2015). Thinking about numbers is not my idea of fun: need for cognition mediates age differences in numeracy performance. Med. Decis. Mak. 35, 22–26. 10.1177/0272989X14542485 25035261

[B11] Bruine de Bruin W. Parker A. M. Fischhoff B. (2012). Explaining adult age differences in decision-making competence. J. Behav. Decis. Mak. 25, 352–360. 10.1002/bdm.712

[B12] Bruine de Bruin W. Strough J. Parker A. M. (2014). Getting older isn’t all that bad: better decisions and coping when facing “sunk costs”. Psychol. Aging 29, 642–649. 10.1037/a0036308 25244483 PMC4362707

[B13] Cacioppo J. T. Petty R. E. Feinstein J. A. Jarvis W. G. (1996). Dispositional differences in cognitive motivation: the life and times of individuals varying in need for cognition. Psychol. Bull. 119, 197–253. 10.1037/0033-2909.119.2.197

[B14] Carstensen L. L. (2006). The influence of a sense of time on human development. Science 312, 1913–1915. 10.1126/science.1127488 16809530 PMC2790864

[B15] Carstensen L. L. Mikels J. A. (2005). At the intersection of emotion and cognition: aging and the positivity effect. Curr. Dir. Psychol. Sci. 14, 117–121. 10.1111/j.0963-7214.2005.00348.x

[B16] Carstensen L. L. Turan B. Scheibe S. Ram N. Ersner-Hershfield H. Samanez-Larkin G. R. (2011). Emotional experience improves with age: evidence based on over 10 years of experience sampling. Psychol. Aging 26, 21–33. 10.1037/a0021285 20973600 PMC3332527

[B17] Charles S. Carstensen L. (2010). Social and emotional aging. Annu. Rev. Psychol. 61, 383–409. 10.1146/annurev.psych.093008.100448 19575618 PMC3950961

[B18] Couper M. P. Singer E. (2009). The role of numeracy in informed consent for surveys. J. Empir. Res. Hum. Res. Ethics 4, 17–26. 10.1525/jer.2009.4.4.17 19919316 PMC2857726

[B19] Covey M. K. McAuley E. Kapela M. C. Collins E. G. Alex C. G. Berbaum M. L. (2012). Upper-body resistance training and self-efficacy enhancement in COPD. J. Pulm. Respir. Med. 20, 1–16. 10.4172/2161-105X.S9-001 24707449 PMC3975911

[B20] Crawford J. D. Stankov L. (1996). Age differences in the realism of confidence in judgments: a calibration study using tests of fluid and crystallized intelligence. Learn. Indiv. Differ. 8, 83–103. 10.1016/S1041-6080(96)90027-8

[B21] Deen D. Lu W. Rothstein D. Santana L. Gold M. R. (2011). Asking questions: the effect of a brief intervention in community health centers on patient activation. Patient Educ. Couns. 84, 257–260. 10.1016/j.pec.2010.07.026 20800414

[B22] Del Missier F. Mäntylä T. Hansson P. Bruine de Bruin W. Parker A. M. Nilsson L. (2013). The multifold relationship between memory and decision making: An individual-differences study. J. Exp. Psychol. Learn. Mem. Cogn. 39, 1344–1364. 10.1037/a0032379 23565790 PMC4160880

[B23] Del Missier F. Mäntylä T. Nilsson L. (2015). “Aging, memory, and decision making,” in Aging and Decision Making: Empirical and Applied Perspectives, eds Hess T. Strough J. Löckenhoff C. (San Diego, CA: Elsevier Academic Press), 127–149. 10.1016/B978-0-12-417148-0.00007-8

[B24] Downs J. S. Bruine de Bruin W. Fischhoff B. Murray P. J. (in press). Behavioral decision research intervention reduces risky sexual behavior. Curr. HIV Res. 10.2174/1570162x13666150511145328 PMC5523954 26149165

[B25] Downs J. S. Murray P. J. Bruine de Bruin W. White J. P. Palmgren C. Fischhoff B. (2004). Interactive video behavioral intervention to reduce adolescent females’ STD risk: a randomized controlled trial. Soc. Sci. Med. 59, 1561–1572. 10.1016/j.socscimed.2004.01.032 15279915

[B26] Eccles J. (2009). Who am I and what am I going to do with my life? Personal and collective identities as motivators of action. Educ. Psychol. 44, 78–89. 10.1080/00461520902832368

[B27] Eccles. J., Wigfield A. (2002). Motivational beliefs, values, and goals. Annu. Rev. Psychol. 53, 109–132. 10.1146/annurev.psych.53.100901.135153 11752481

[B28] Elliot A. J. Conroy D. E. Barron K. E. Murayama K. (2010). “Achievement motives and goals: a developmental analysis,” in The Handbook of Life-span Development, Vol. 2, *Social and Emotional Development*, eds Lamb M. E. Freund A. M. Lerner R. M. (Hoboken, NJ: John Wiley & Sons Inc), 474–510.

[B29] Ennis G. E. Hess T. M. Smith B. T. (2013). The impact of age and motivation on cognitive effort: implications for cognitive engagement in older adulthood. Psychol. Aging 28, 495–504. 10.1037/a0031255 23421325 PMC3788706

[B30] Finucane M. L. Gullion C. M. (2010). Developing a tool for measuring the decision-making competence of older adults. Psychol. Aging 25, 271–288. 10.1037/a0019106 20545413 PMC2918639

[B31] Frederickson B. L. (2001). The role of positive emotions in positive psychology: the broaden-and-build theory of positive emotions. Am. Psychol. 56, 218–226. 10.1037/0003-066X.56.3.218 11315248 PMC3122271

[B32] Frosch D. L. Rincon D. Ochoa S. Mangione C. M. (2010). Activating seniors to improve chronic disease care: results from a pilot intervention study. J. Am. Geriatr. Soc. 58, 1496–1503. 10.1111/j.1532-5415.2010.02980.x 20662953 PMC2955177

[B33] Fung H. H. Carstensen L. (2003). Sending memorable messages to the old: age differences in preferences and memory for advertisements. J. Pers. Soc. Psychol. 85, 163–178. 10.1037/0022-3514.85.1.163 12872892

[B34] Fung H. H. Carstensen L. L. Lutz A. M. (1999). The influence of time on social preferences: implications for life-span development. Psychol. Aging 14, 595–604. 10.1037/0882-7974.14.4.595 10632147

[B35] Gigerenzer G. Hoffrage U. Kleinbölting H. (1991). Probabilistic mental models: a Brunswikian theory of confidence. Psychol. Rev. 98, 506–528. 10.1037/0033-295X.98.4.506 1961771

[B36] Gentner D. (2002). “Mental models, psychology of,” in International Encyclopedia of the Social and Behavioral Sciences, eds Smelser N. J. Bates P. B. (Amsterdam: Elsevier), 9683–9687.

[B37] Greene J. Hibbard J. H. (2012). Why does patient activation matter? An examination of the relationships between patient activation and health-related outcomes. J. Gen. Intern. Med. 27, 520–526. 10.1007/s11606-011-1931-2 22127797 PMC3326094

[B38] Hansson P. Rönnlund M. Juslin P. Nilsson L. (2008). Adult age differences in the realism of confidence judgments: overconfidence, format dependence, and cognitive predictors. Psychol. Aging 23, 531–544. 10.1037/a0012782 18808243

[B39] Hess T. M. (2005). Memory and Aging in Context. Psychol. Bull. 131, 383–406. 10.1037/0033-2909.131.3.383 15869334

[B40] Hess T. M. (2014). Selective engagement of cognitive resources: Motivational influences on older adults’ cognitive functioning. Perspect. Psychol. Sci. 9, 388–407. 10.1177/1745691614527465 PMC5911399 26173272

[B41] Hess T. M. Germain C. M. Swaim E. L. Osowski N. L. (2009). Aging and selective engagement: The moderating impact of motivation on older adults’ resource utilization. J. Gerontol. B Psychol. Sci. Soc. Sci. 64B, 447–456. 10.1093/geronb/gbp020 19357075 PMC2697500

[B42] Hess T. M. Queen T. L. (2014). “Aging influences on judgment and decision processes: interactions between ability and experience,” in Emotion, Social Cognition, and Problem Solving During Adulthood, eds Verhaeghen P. Hertzog C. (New York, NY: Oxford University Press), 238–255.

[B43] Hess T. M. Queen T. L. Ennis G. E. (2013). Age and self-relevance effects on information search during decision making. J. Gerontol. B Psychol. Sci. Soc. Sci. 68, 703–711. 10.1093/geronb/gbs108 23197342 PMC3859358

[B44] Hess T. M. Rosenberg D. C. Waters S. J. (2001). Motivation and representational processes in adulthood: the effects of social accountability and information relevance. Psychol. Aging 16, 629–642. 10.1037/0882-7974.16.4.629 11766917

[B45] Hibbard J. H. Mahoney E. R. (2010). Toward a theory of patient and consumer activation. Patient Educ. Couns. 78, 377–381. 10.1016/j.pec.2009.12.015 20188505

[B46] Hibbard J. H. Mahoney E. Stockard J. Tusler M. (2005). Development and testing of a short form of the Patient Activation Measure (PAM). Health Serv. Res. 40, 1918–1930. 10.1111/j.1475-6773.2005.00438.x 16336556 PMC1361231

[B47] Hibbard J. H. Stockard J. Mahoney E. R. Tusler M. (2004). Development of the Patient Activation Measure (PAM): conceptualizing and measuring activation in patient and consumers. Health Serv. Res. 39, 1005–1026. 10.1111/j.1475-6773.2004.00269.x 15230939 PMC1361049

[B48] Hummert M. (2011). “Age stereotypes and aging” in Handbook of the Psychology of Aging, 7th Edn, eds Schaie K. Willis S. L. (San Diego, CA: Elsevier Academic Press), 249–262.

[B49] Isaacowitz D. M. Choi Y. (2012). Looking, feeling, and doing: are there age differences in attention, mood, and behavioral responses to skin cancer information? Health Psychol. 31, 650–659. 10.1037/a0026666 22149125 PMC3360113

[B50] Isen A. (2008). “Some ways in which positive affect influences decision making and problem solving,” in Handbook of Emotions, Vol. 3, eds Lewis M. Haviland-Jones J. M. Barrett L. F. (New York, NY: Guilford Press), 548–573.

[B51] Johnson M. M. S. (1990). Age differences in decision making: a process methodology for examining strategic information processing. J. Gerontol. B Psychol. Sci. Soc. Sci. 45, 75–78. 10.1093/geronj/45.2.P75 2179392

[B52] Juslin P. Winman A. Olsson H. (2000). Naive empiricism and dogmatism in confidence research: a critical examination of the hard-easy effect. Psychol. Rev. 107, 384–396. 10.1037/0033-295X.107.2.384 10789203

[B53] Keren G. (1991). Calibration and probability judgments: conceptual and methodological issues. Acta Psychol. 3, 217–273. 10.1016/0001-6918(91)90036-Y

[B54] Kim S. Healey M. K. Goldstein D. Hasher L. Wiprzycka U. J. (2008). Age differences in choice satisfaction: a positivity effect in decision making. Psychol. Aging 23, 33–38. 10.1037/0882-7974.23.1.33 18361652

[B55] Kinsella K. He W. (2008). An Aging World: 2008. International Population Reports, P95/09-1. Washington, DC: US Government Printing Office.

[B56] Kovalchik S. Camerer C. F. Grether D. M. Plott C. R. Allman J. M. (2005). Aging and decision making: a comparison between neurologically healthy elderly and young individuals. J. Econ. Behav. Organ. 58, 79–94. 10.1016/j.jebo.2003.12.001

[B57] Kullgren J. T. Harkins K. A. Bellamy S. L. Gonzales A. Tao Y. Zhu J. (2014). A mixed-methods randomized controlled trial of financial incentives and peer networks to promote walking among older adults. Health Educ. Behav. 41, 43S–50S. 10.1177/1090198114540464 25274710 PMC4382353

[B58] Larson J. L. Covey M. K. Kapella M. C. Alex C. G. McAuley E. (2014). Self-efficacy enhancing intervention increases light physical activity in people with chronic obstructive pulmonary disease. Int. J. Chron. Obstruct. Pulmon. Dis. 9, 1081–1090. 10.2147/COPD.S66846 25336939 PMC4199844

[B59] Leventhal E. A. Leventhal H. Schaefer P. Easterling D. (1993). Conservation of energy, uncertainty reduction, and swift utilization of medical care among the elderly. J. Gerontol. B Psychol. Sci. Soc. Sci. 48, 78–86. 10.1093/geronj/48.2.P78 8473701

[B60] Levy B. R. Zonderman A. B. Slade M. D. Ferrucci L. (2012). Memory shaped by age stereotypes over time. J. Gerontol. B Psychol. Sci. Soc. Sci. 67B, 432–436. 10.1093/geronb/gbr120 22056832 PMC3391075

[B61] Li Y. Gao J. Enkavi A. Z. Zaval L. Weber E. U. Johnson E. J. (2014). Sound credit scores and financial decisions despite cognitive aging. Proc. Natl. Acad. Sci. U.S.A. 112, 65–69. 10.1073/pnas.1413570112 25535381 PMC4291658

[B62] Löckenhoff C. E. Carstensen L. L. (2007). Aging, emotion, and health-related decision strategies: motivational manipulations can reduce age differences. Psychol. Aging 22, 134–146. 10.1037/0882-7974.22.1.134 17385990

[B63] Lorig K. Green M. Ritter P. L. Jernigan V. B. B. Laurent D. D. (2009). Online diabetes self-management program: a randomized study. Diabetes Care 33, 1275–1281. 10.2337/dc09-2153 20299481 PMC2875437

[B64] Mather M. Carstensen L. L. (2005). Aging and motivated cognition: the positivity effect in attention and memory. Trends Cogn. Sci. 9, 496–502. 10.1016/j.tics.2005.08.005 16154382

[B65] Mather M. Knight M. McCaffrey M. (2005). The allure of the alignable: younger and older adults’ false memories of choice features. J. Exp. Psychol. Gen. 134, 38–51. 10.1037/0096-3445.134.1.38 15702962

[B66] McAuley E. Courneya K. S. Rudolph D. L. Lox C. L. (1994). Enhancing exercise adherence in middle-aged males and females. Prev. Med. 23, 498–506. 10.1006/pmed.1994.1068 7971878

[B67] Mikels J. A. Löckenhoff C. E. Maglio S. J. Carstensen L. L. Goldstein M. K. Garber A. (2010). Following your heart or your head: focusing on emotions versus information differentially influences the decisions of younger and older adults. J. Exp. Psychol. Appl. 16, 87–95. 10.1037/a0018500 20350046 PMC3919140

[B68] Mikels J. A. Shuster M. M. Thai S. T. (2015). “Aging, emotion, and decision making,” in Aging and Decision Making: Empirical and Applied Perspectives, eds Hess T. M. Strough J. Löckenhoff C. (San Diego, CA: Elsevier Academic Press), 169–188.

[B69] Miron-Shatz T. Hanoch Y. Doniger G. M. Omer Z. B. Ozanne E. M. (2014). Subjective but not objective numeracy influences willingness to pay for BRCA1/2 genetic testing. Judgm. Decis. Mak. 9, 152–158.

[B70] Meyer B. F. Talbot A. P. Ranalli C. (2007). Why older adults make more immediate treatment decisions about cancer than younger adults. Psychol. Aging 22, 505–524. 10.1037/0882-7974.22.3.505 17874951

[B71] Meyer D. Leventhal H. Gutmann M. (1985). Common-sense models of illness: The example of hypertension. Health Psychol. 4, 115–135. 4018002 10.1037//0278-6133.4.2.115

[B72] Morgan M. G. Fischhoff B. Bostrom A. Atman C. J. (2002). Risk Communication: a Mental Models Approach. Cambridge: Cambridge University Press.

[B73] Nersessian N. J. (1992). Cognitive Models of Science, eds Giere R. N. (Minneapolis: Univ of Minnesota Press), 3–45.

[B74] Noar S. M. (2005). A health educator’s guide to theories of health behavior. Int. Q. Community Health Educ. 24, 75–92. 10.2190/DALP-3F95-GCT3-M922 17690053

[B75] Notthoff N. Carstensen L. L. (2014). Positive messaging promotes walking in older adults. Psychol. Aging 29, 329–341. 10.1037/a0036748 24956001 PMC4069032

[B76] Parker A. M. Bruine de Bruin W. Yoong J. Willis R. (2012). Inappropriate confidence and retirement planning: four studies with a national sample. J. Behav. Decis. Mak. 25, 382–389. 10.1002/bdm.745 23049164 PMC3462465

[B77] Parker A. M. Stone E. R. (2014). Identifying the effects of unjustified confidence versus overconfidence: Lessons learned from two analytic methods. J. Behav. Decis. Mak. 27, 134–145. 10.1002/bdm.1787 25309037 PMC4191861

[B78] Payne B. R. Jackson J. J. Hill P. L. Gao X. Roberts B. W. Stine-Morrow E. L. (2012). Memory self-efficacy predicts responsiveness to inductive reasoning training in older adults. J. Gerontol. B Psychol. Sci. Soc. Sci. 67B, 27–35. 10.1093/geronb/gbr073 21743037 PMC3267022

[B79] Peters E. Bjalkebring P. (2015). Multiple numeric competencies: when a number is not just a number. J. Pers. Soc. Psychol. 108, 802–822. 10.1037/pspp0000019 25285966

[B80] Peters E. Bruine de Bruin W. (2012). “Aging and decision skills,” in Judgment and Decision Making as a Skill: Learning, Development, and Evolution, eds Dhami M. K. Schlottmann A. Waldmann M. (New York: Cambridge University Press), 113–139.

[B81] Peters E. Hess T. M. Västfjäll D. Auman C. (2007). Adult age differences in dual information processes: implications for the role of affective and deliberative processes in older adults’ decision making. Perspect. Psychol. Sci. 2, 1–23. 10.1111/j.1745-6916.2007.00025.x 26151915

[B83] Reed A. E. Chan L. Mikels J. A. (2014a). Meta-analysis of the age-related positivity effect: age differences in preferences for positive over negative information. Psychol. Aging 29, 1–15. 10.1037/a0035194 24660792

[B84] Reed A. E. Sims T. L. English T. Carstensen L. (2014b). “The age-related positivity effect in decision making: implications for choice quality,” in Age Differences in Decision Making: Balancing Affect and Reason in Financial and Healthcare Settings. Symposium Conducted at the Annual Meeting of the Gerontological Society of America, eds Strough J. Löckenhoff C. E. (Chairs), (Gerontological Society of America: Washington).

[B85] Reed A. E. Mikels J. A. Löckenhoff C. E. (2013). Preferences for choice across adulthood: age trajectories and potential mechanisms. Psychol. Aging 28, 625–632. 10.1037/a0031399 23437900

[B86] Reed A. E. Mikels J. A. Simon K. I. (2008). Older adults prefer less choice than young adults. Psychol. Aging 23, 671–675. 10.1037/a0012772 18808256 PMC2631411

[B87] Reyna V. F. Nelson W. L. Han P. K. Dieckmann N. F. (2009). How numeracy influences risk comprehension and medical decision making. Psychol. Bull. 135, 943–973. 10.1037/a0017327 19883143 PMC2844786

[B88] Rolison J. J. Wood S. Hanoch Y. Liu P. J. (2013). Subjective numeracy scale as a tool for assessing statistical numeracy in older adult populations. Gerontology 59, 283–288. 10.1159/000345797 23391745

[B89] Shamaskin A. M. Mikels J. A. Reed A. E. (2010). Getting the message across: age differences in the positive and negative framing of health care messages. Psychol. Aging 25, 746–751. 10.1037/a0018431 20677886

[B90] Smith S. G. Wolf M. S. von Wagner C. (2010). Socioeconomic status, statistical confidence, and patient-provider communication: an analysis of the Health Information National Trends Survey (HINTS 2007). J. Health Commun. 15(Suppl. 3), 169–185. 10.1080/10810730.2010.522690 21154092

[B91] Strough J. Karns T. Schlosnagle L. (2011a). Decision making heuristics and biases across the life span. Ann. N. Y. Acad. Sci. 1235, 57–74. 10.1111/j.1749-6632.2011.06208.x 22023568 PMC3755606

[B92] Strough J. Schlosnagle L. DiDonato L. (2011b). Understanding decisions about sunk costs from older and younger adults’ perspectives. J. Gerontol. B Psychol. Sci. Soc. Sci. 66B, 681–686. 10.1093/geronb/gbr057 21765061

[B93] Strough J. Mehta C. M. McFall J. P. Schuller K. L. (2008). Are older adults less subject to the sunk-cost fallacy than younger adults? Psychol. Sci. 19, 650–652. 10.1111/j.1467-9280.2008.02138.x 18727779

[B94] Strough J. Parker A. M. Bruine de Bruin W. (2015). “Understanding life-span developmental changes in decision-making competence,” in Aging and Decision Making: Empirical and Applied Perspectives, eds Hess T. Strough J. Löckenhoff C. (San Diego, CA: Elsevier Academic Press), 235–257.

[B95] Thomas A. K. Millar P. R. (2012). Reducing the framing effect in older and younger adults by encouraging analytic processing. J. Gerontol. B Psychol. Sci. Soc. Sci. 67B, 139–149. 10.1093/geronb/gbr076 21964668

[B96] von Helversen B. Mata R. (2012). Losing a dime with a satisfied mind: positive affect predicts less search in sequential decision making. Psychol. Aging 27, 825–839. 10.1037/a0027845 22449028

[B97] Vosniadou S. (2002). “Model-based reasoning in conceptual development.” in Model-based reasoning., eds Magnani L. Nersessian N. J. (New York: Springer), 353–368.

[B98] Vosniadou S. Brewer W. F. (1992). Mental models of the earth: a study of conceptual change in childhood. Cogn. Psychol. 24, 535–585. 10.1016/0010-0285(92)90018-W

[B99] West R. L. Hastings E. C. (2011). Self-regulation and recall: growth curve modeling of intervention outcomes for older adults. Psychol. Aging 26, 803–812. 10.1037/a0023784 21604891 PMC3801089

[B100] Wilson T. D. Schooler J. W. (1991). Thinking too much: introspection can reduce the quality of preferences and decisions. J. Pers. Soc. Psychol. 60, 181–192. 10.1037/0022-3514.60.2.181 2016668

[B101] Woodward N. J. Wallston B. S. (1987). Age and health care beliefs: self-efficacy as a mediator of low desire for control. Psychol. Aging 2, 3–8. 10.1037/0882-7974.2.1.3 3268188

[B102] Worthy D. A. Otto A. R. Doll B. B. Byrne K. A. Maddox W. T. (2015). Older adults are highly responsive to recent events during decision-making. Decisions 2, 27–38. 10.1037/dec0000018 25580469 PMC4288013

[B103] Zaval L. Li Y. Johnson E. Weber E. (2015). “Complementary contributions of fluid and crystallized intelligence to decision making across the life span,” in Aging and Decision Making: Empirical and Applied Perspectives, eds Hess T. Strough J. Löckenhoff C. (San Diego, CA: Elsevier Academic Press), 149–168.

